# Supplementary material for: The spatial transcriptomic landscape of the healing mouse intestine following damage
Source: Nat Commun. 2022 Feb 11;13:828. doi: 10.1038/s41467-022-28497-0 (PMC8837647; doi:10.1038/s41467-022-28497-0)
Supplement: Supplementary file 1 — Supplementary information [file 41467_2022_28497_MOESM1_ESM.pdf]

Supplementary material

“The spatial transcriptomic landscape of the healing mouse intestine following damage”

Sara M. Parigi[1,2,*], Ludvig Larsson[3,*], Srustidhar Das[1,2,*], Ricardo O. Ramirez Flores[4], Annika Frede[1,2], Kumar P. Tripathi[1,2], Oscar E. Diaz[1,2], Katja Selin[1,2], Rodrigo A. Morales[1,2], Xinxin Luo[1,2], Gustavo Monasterio[1,2], Camilla Engblom[5], Nicola Gagliani[1,2,6], Julio Saez-Rodriguez[4], Joakim Lundeberg[3] and Eduardo J. Villablanca[1,2]

[1] Division of Immunology and Allergy, Department of Medicine Solna, Karolinska Institute and University Hospital, Stockholm, Sweden

[2] Center of Molecular Medicine, Stockholm, Sweden

[3] Science for Life Laboratory, Department of Gene Technology, KTH Royal Institute of Technology, Stockholm, Sweden

[4] Heidelberg University, Faculty of Medicine, and Heidelberg University Hospital, Institute for Computational Biomedicine, Bioquant, Heidelberg, Germany

[5] Department of Cell and Molecular Biology, Karolinska Institute, Stockholm, Sweden

[6] I. Department of Medicine and Department of General, Visceral and Thoracic Surgery, University Medical Center Hamburg-Eppendorf, Hamburg, Germany

[*]These authors contributed equally

Correspondence should be addressed to E.J.V. (eduardo.villablanca@ki.se).

Supplementary Fig. 1

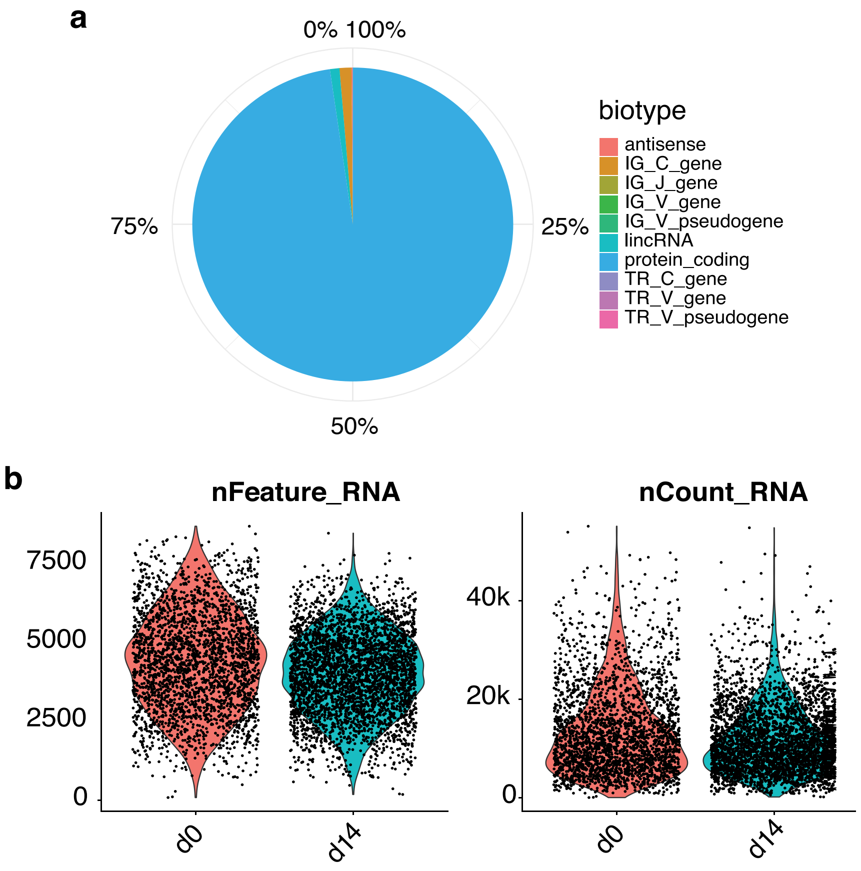

Supplementary Fig. 1, Overview of the quality control of the ST datasets.

(a) Pie chart of pre-filtered dataset showing percentages of different RNA biotypes.

(b) Violin plots showing the number of unique genes per spot (nFeature_RNA) and RNA copy count (nCount_RNA) in d0 (from Figure 1) and d14 sample (from figure 3). IG: immunoglobulin; lincRNA: long intervening non-coding RNA; TR: T cell receptor.

Supplementary Fig. 2

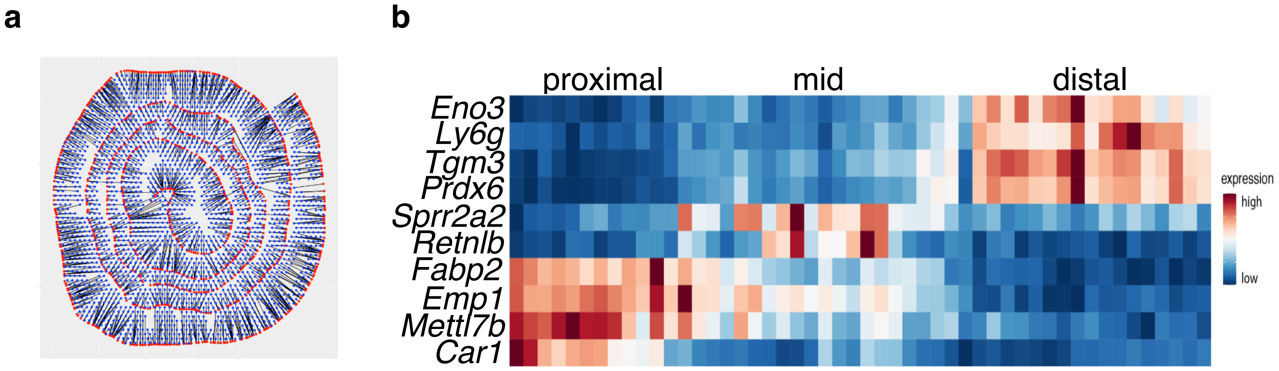

Supplementary Fig. 2, Digital unrolling of the colon Swiss roll and regional distribution of gene expression along the colon.

(a) Schematic representation displaying the colon digital unrolling strategy. Red ST spots were used as a bottom coordinate and represent the base of the unrolled colon. All the other ST spots (shown in blue) were aligned to the red spots according to the lines shown in the figure.

(b) Heatmap showing expression of the indicated genes in proximal, mid and distal colon.

Supplementary Fig. 3

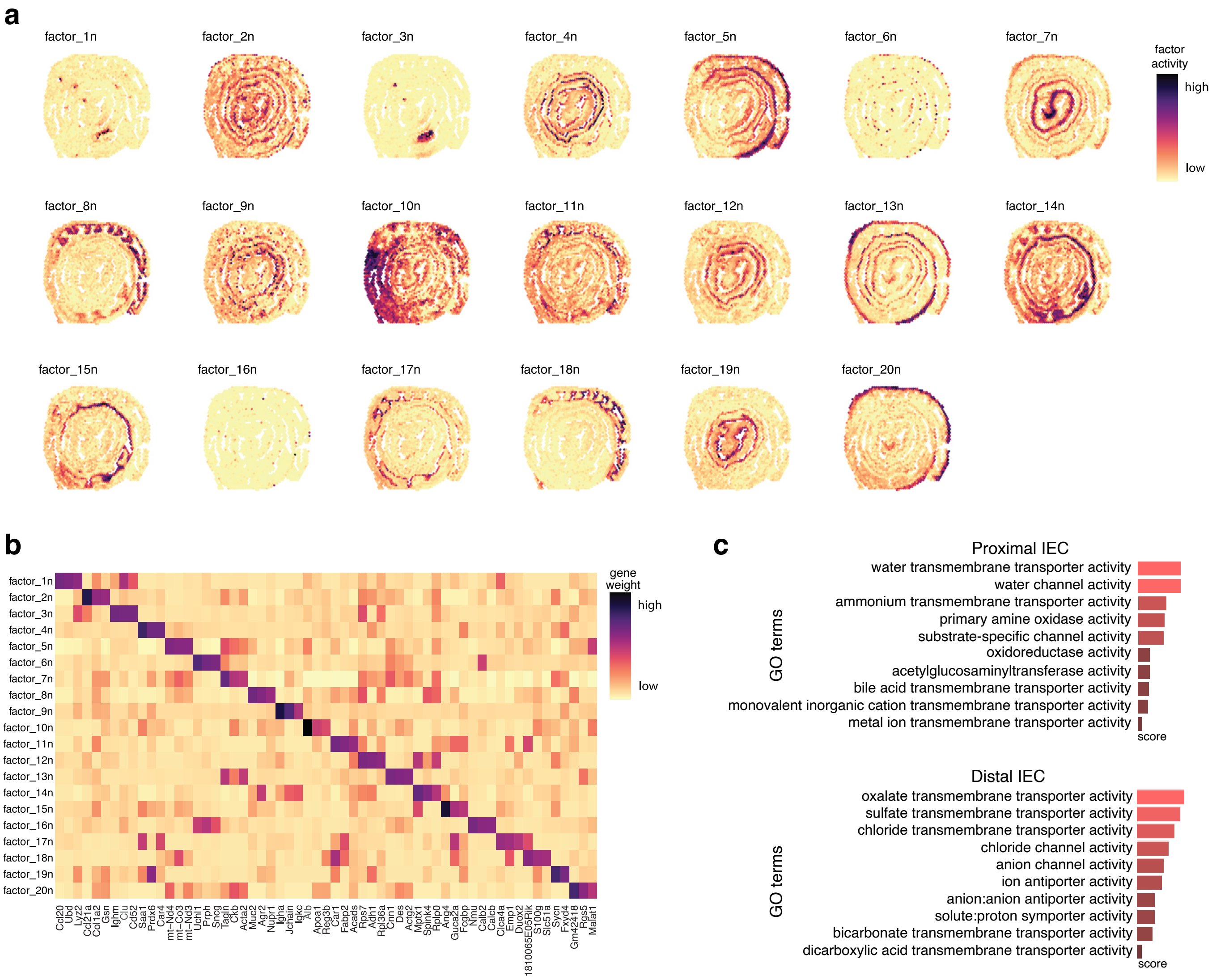

Supplementary Fig. 3, Non-negative matrix factorization of steady state colon.

(a) ModuleScore representation of the distribution of 20 Factors identified in steady state (naive) colon. Each dot represents a ST spot and is color-coded based on an enrichment score (high: black, low: yellow) for each Factor gene signature.

(b) Heatmap showing the top 2-3 genes in each of the 20 Factor identified in the d0 colon.

(c) Gene ontology (GO) annotation showing the top enriched pathways in Proximal IEC (factors 8n, 11n, 17n and 18n) and distal IEC (factors 4n and 19n).

Supplementary Fig. 4

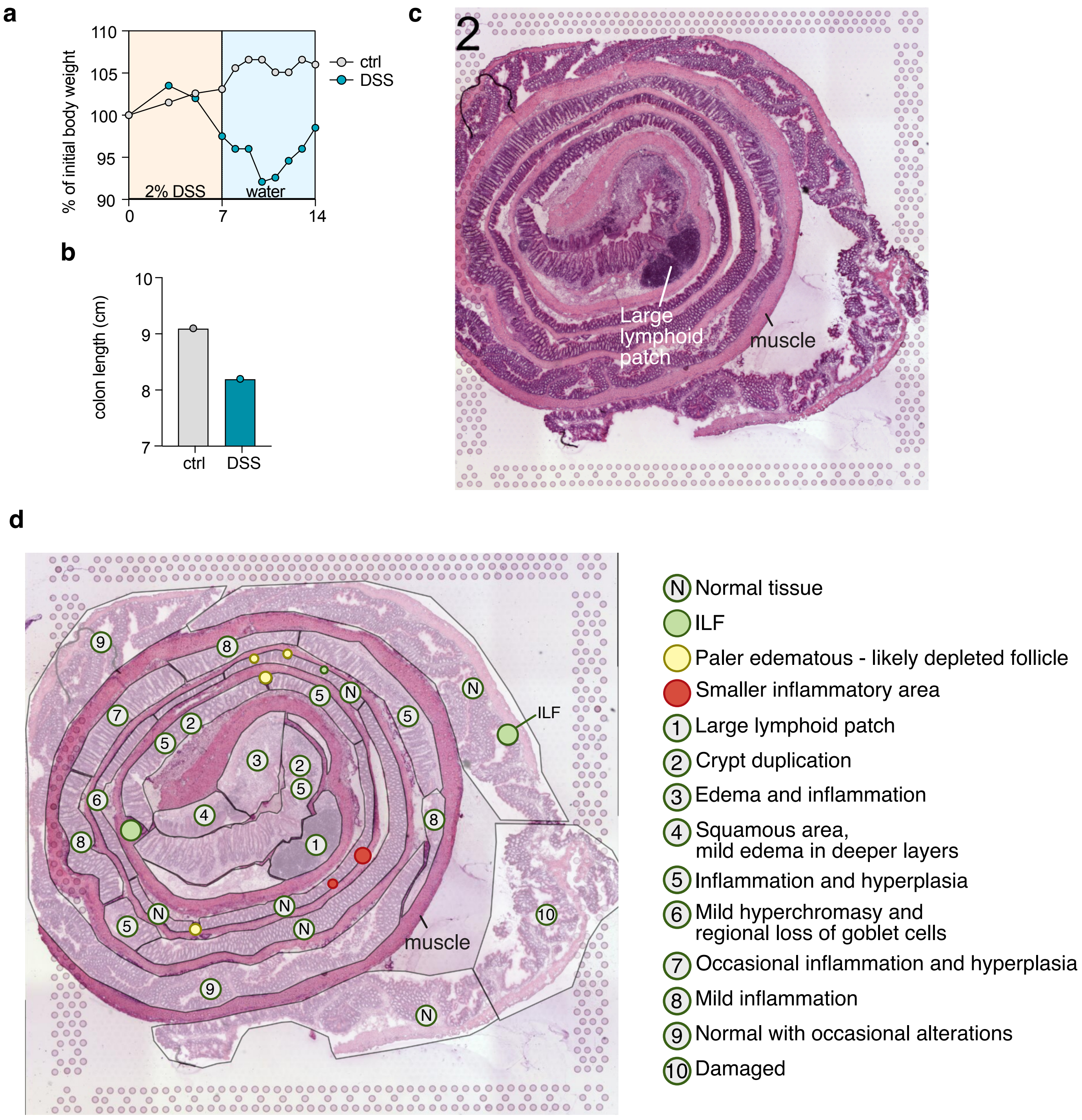

Supplementary Fig. 4, Macroscopic and histological disease score upon DSS-treatment.

(a) Graph showing percentage of initial body weight in the control (ctrl) mouse (i.e. colon d0, administered with regular drinking water for 14 days) and in the DSS-treated mouse (i.e. colon d14, administered 2%DSS in drinking water for 7 days followed by 7 days of regular water).

(b) Length in cm of the colonic tissue at day14 in ctrl and DSS-treated mouse.

(c) Hematoxylin and eosin staining of the colonic Swiss roll on d14. Macroscopically visible structures (e.g. lymphoid follicle and muscle) are indicated.

(d) Hematoxylin and eosin representation of colon d14 where distinct areas have been outlined and categorized based on the blinded pathologist' scoring. Each area has been labeled with a number, color or letter based on the legend on the right. ILF: isolated lymphoid follicle.

Supplementary Fig. 5

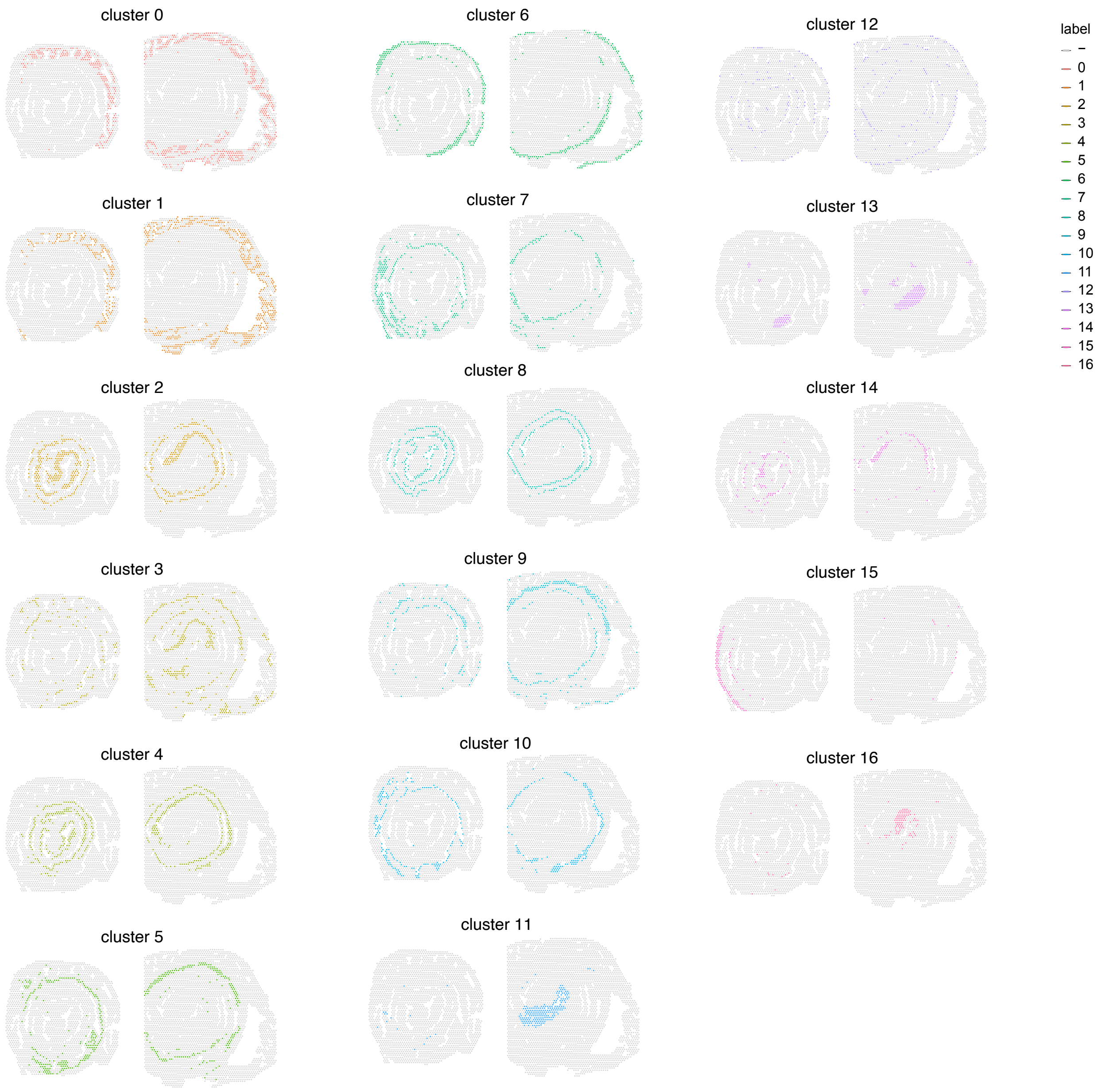

Supplementary Fig. 5, Cluster analysis of d0 and d14 colon.

Schematic representation of the 17 clusters distribution (from Figure 3c) in colon d0 (on the left) and d14 (on the right). Each ST spot has been color coded and assigned to a unique cluster.

Supplementary Fig. 6

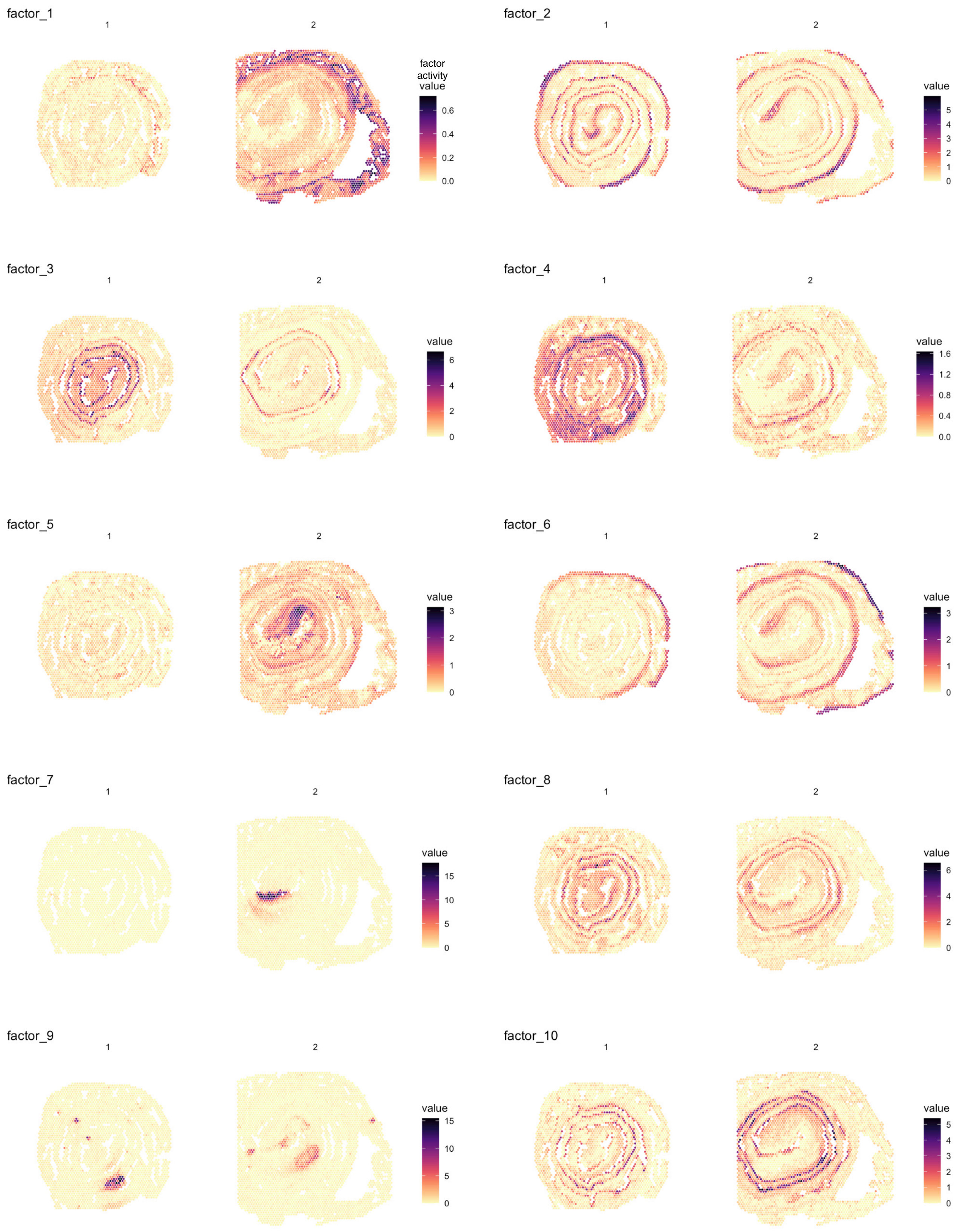

Supplementary Fig. 7

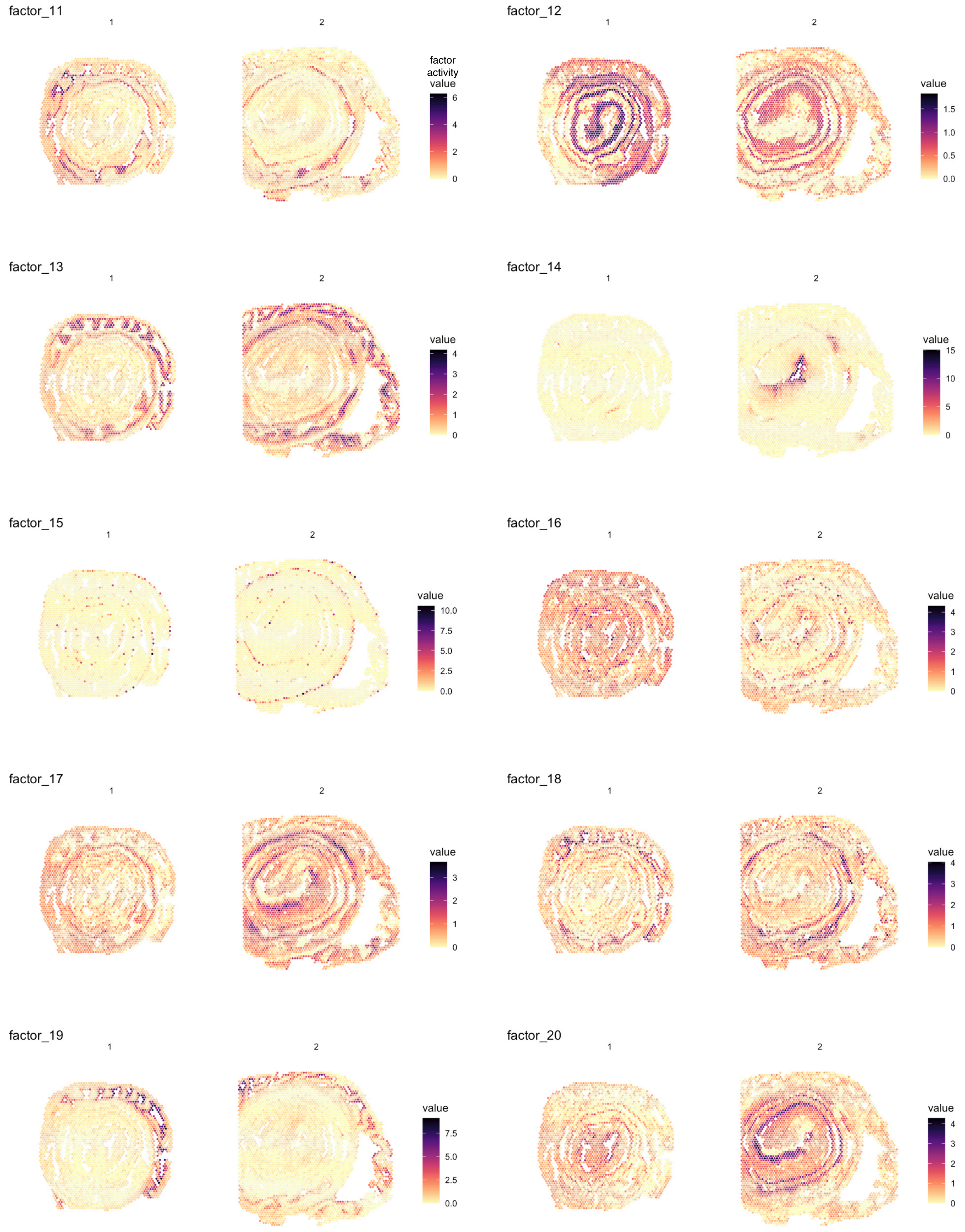

Supplementary Fig. 6 and 7, Non-negative matrix factorization of colon at d0 and d14.

ModuleScore representation of the distribution of 20 Factors identified in steady state (d0, on the left) and healing colon (d14, on the right). Each dot represents a ST spot and is color-coded based on an enrichment score (high: black, low: yellow) for each Factor gene signature.

Supplementary Fig. 8

a

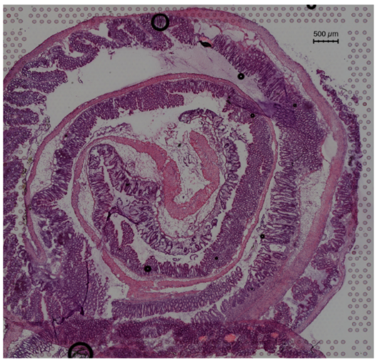

b

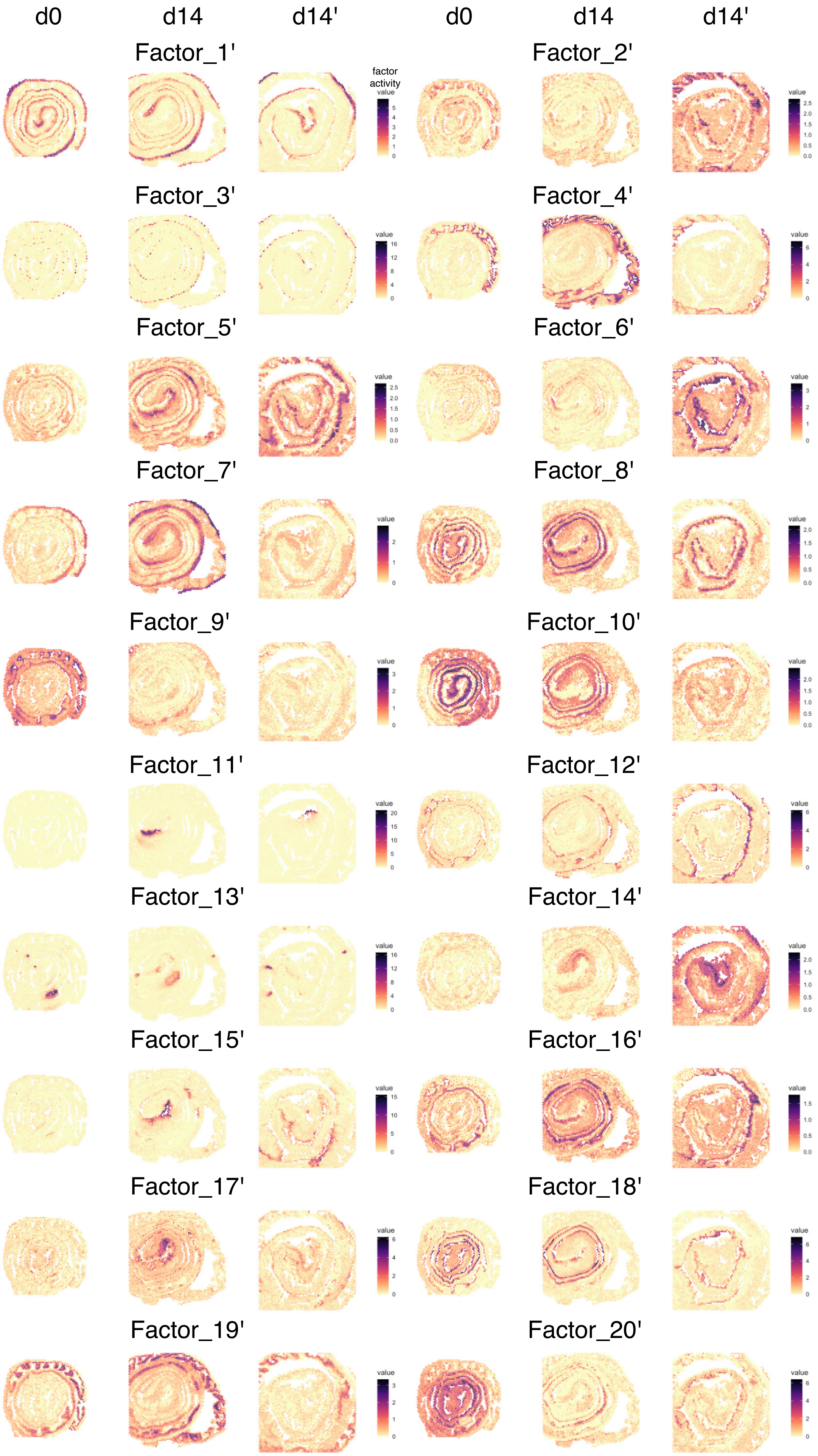

Supplementary Fig. 8, Non-negative matrix factorization of colon d0 and two replicates of colon d14.

(a) Hematoxylin and eosin representation of the biological replicate of colon d14'.

(b) ModuleScore representation of the distribution of 20 Factors identified by reanalyzing the same d0 and d14 from Supplementary Fig.6-7 (named as d0 and d14) and one additional d14 sample (named as d14'). Each dot represents a ST spot and is color-coded based on an enrichment score (high: black, low: yellow) for each Factor gene signature.

Supplementary Fig. 9

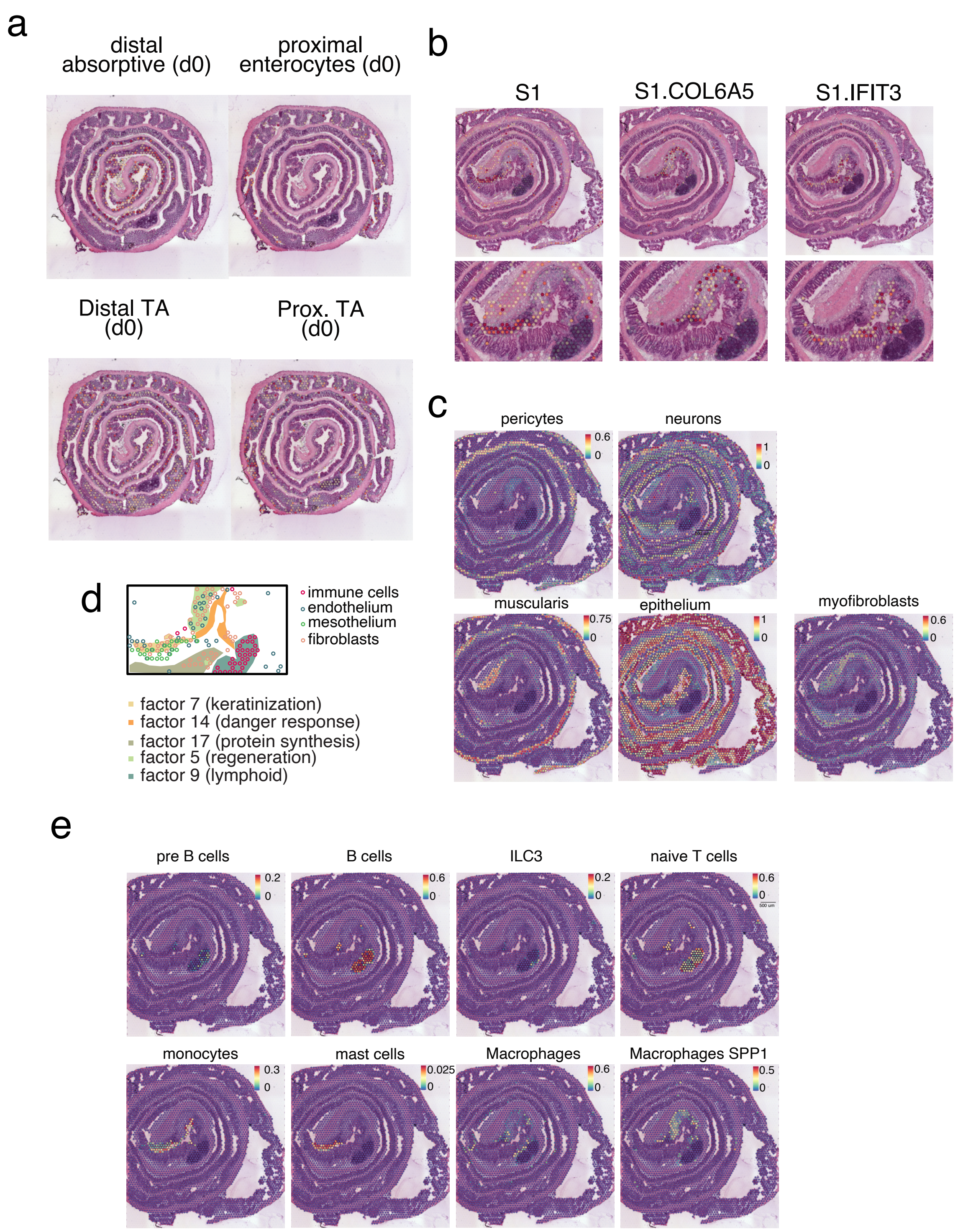

Supplementary Fig. 9, Integration of human scRNAseq onto murine spatial transcriptomic dataset.

(a) Mapping of scRNAseq signature of distal absorptive or proximal enterocytes and distal and proximal transit amplifying (TA) cells on murine d0 ST dataset. ST spots are displayed on the Hematoxylin and eosin background and yellow-red spots are enriched for the indicated scRNAseq signature.

(b) Mapping of scRNAseq signature of S1, S1-COL6A5 and S1-IFIT3 on murine d14 ST dataset.

(c) Integration of the indicated human intestinal cell transcriptomic profile onto the murine Visium dataset at day 14.

(d) Schematic summary of the distribution and overlap between human intestinal cell signature (empty circles) and murine factor (filled squares).

(e) Integration of the indicated human immune cell subsets transcriptomic onto the murine Visium dataset at day 14.

Supplementary Fig. 10

a

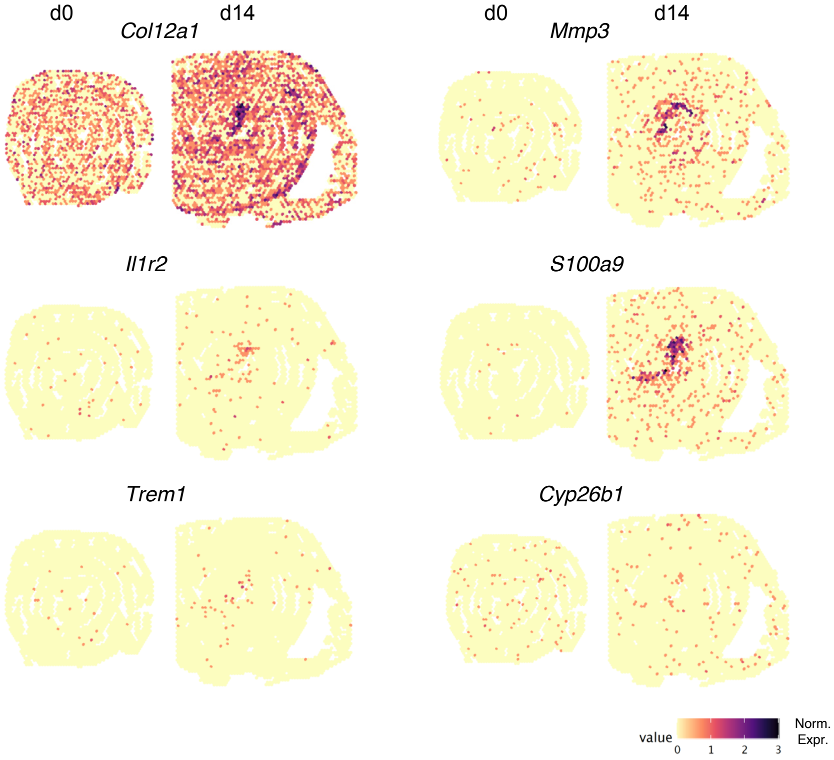

b

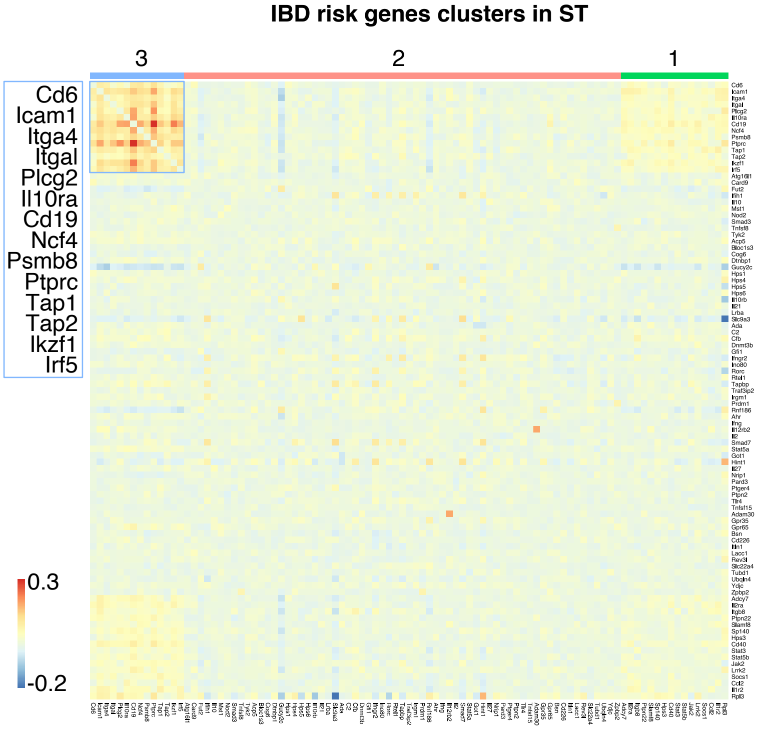

Supplementary Fig. 10, Integration of Ulcerative colitis signature and IBD risk genes onto murine spatial transcriptomic (ST) dataset.

(a) ModuleScore representation of the distribution of the indicated UC1 genes in steady state (d0, on the left) and healing colon (d14, on the right). Each dot represents a ST spot and is color-coded based on an enrichment score (high: black, low: yellow) for each gene.

(b) Heatmap showing the spatial expression correlation between Inflammatory Bowel Disease (IBD) risk genes. IBD risk variants are clustered based on their similar spatial expression pattern. Outlined by a blue box are the genes belonging to cluster 3.
