# Supplementary material for: The spatial transcriptomic landscape of the healing mouse intestine following damage
Source: Nat Commun. 2022 Feb 11;13:828. doi: 10.1038/s41467-022-28497-0 (PMC8837647; doi:10.1038/s41467-022-28497-0)
Supplement: Supplementary file 3 — Description of Additional Supplementary Files [file 41467_2022_28497_MOESM3_ESM.docx]

Description of Additional Supplementary Files

Title: Supplementary Data 1

Description: List of DEGs identified in the clusters from colon d0 and d14. The list of genes in each cluster is shown in separate sheets and the different time points are indicated in a separate column.
