# Supplementary material for: The spatial transcriptomic landscape of the healing mouse intestine following damage
Source: Nat Commun. 2022 Feb 11;13:828. doi: 10.1038/s41467-022-28497-0 (PMC8837647; doi:10.1038/s41467-022-28497-0)
Supplement: Supplementary file 5 — Reporting Summary [file 41467_2022_28497_MOESM5_ESM.pdf]

nature portfolio

Corresponding author(s): Eduardo Villablanca

Last updated by author(s): Dec 14, 2021

Reporting Summary

Nature Portfolio wishes to improve the reproducibility of the work that we publish. This form provides structure for consistency and transparency in reporting. For further information on Nature Portfolio policies, see our Editorial Policies and the Editorial Policy Checklist.

Statistics

For all statistical analyses, confirm that the following items are present in the figure legend, table legend, main text, or Methods section.

n/a	Confirmed	
☐	☒	The exact sample size (n) for each experimental group/condition, given as a discrete number and unit of measurement
☐	☒	A statement on whether measurements were taken from distinct samples or whether the same sample was measured repeatedly
☐	☒	The statistical test(s) used AND whether they are one- or two-sided<br>*Only common tests should be described solely by name; describe more complex techniques in the Methods section.*
☐	☒	A description of all covariates tested
☐	☒	A description of any assumptions or corrections, such as tests of normality and adjustment for multiple comparisons
☐	☒	A full description of the statistical parameters including central tendency (e.g. means) or other basic estimates (e.g. regression coefficient) AND variation (e.g. standard deviation) or associated estimates of uncertainty (e.g. confidence intervals)
☐	☒	For null hypothesis testing, the test statistic (e.g. F, t, r) with confidence intervals, effect sizes, degrees of freedom and P value noted<br>*Give P values as exact values whenever suitable.*
☒	☐	For Bayesian analysis, information on the choice of priors and Markov chain Monte Carlo settings
☐	☒	For hierarchical and complex designs, identification of the appropriate level for tests and full reporting of outcomes
☐	☒	Estimates of effect sizes (e.g. Cohen's d, Pearson's r), indicating how they were calculated

Our web collection on statistics for biologists contains articles on many of the points above.

Software and code

Policy information about availability of computer code

Data collection	The data were generated using kits from 10X genomics and sequenced on a NovaSeq S1 flow cell (Illumina), at a depth of 196-259 million reads per sample, with 28 bases from read 1 and 120 bases from read 2. Leica DM5500 B microscope and Leica Application Suite X (LAS X) was used to acquire tile scans of the entire array and merge images. Metafer Slide Scanning Platform (Metasystems) was used to acquire the fluorescent footprint.
Data analysis	ABI 7500 Software 2.3, Microsoft Office Standard (version 16.54), GraphPad Prism (version 9.0.1), spaceranger command line tool (version 1.0.0, 10X Genomics).<br>Rstudio (version 4.0.0) with the following packages: InputFromTable (STUtility), kNN, dbscan, igraph, gprofiler2, sctransform, Seurat (version 3.1.4.9), biomaRt and fgsea. The following codes were generated and used for this study:Code for PROGENy analysis is available at https://github.com/saezlab/visium_colon_si. Code to explore the healing colon ST datasets is available at https://github.com/ludvigla/murine_colon_explorer and https://github.com/ludvigla/healing_intestine_analysis

For manuscripts utilizing custom algorithms or software that are central to the research but not yet described in published literature, software must be made available to editors and reviewers. We strongly encourage code deposition in a community repository (e.g. GitHub). See the Nature Portfolio guidelines for submitting code & software for further information.

Data

Policy information about availability of data

All manuscripts must include a data availability statement. This statement should provide the following information, where applicable:

- Accession codes, unique identifiers, or web links for publicly available datasets
- A description of any restrictions on data availability
- For clinical datasets or third party data, please ensure that the statement adheres to our policy

The datasets generated during this study have been deposited in theare available at Gene Expression Omnibus (GEO) database underwith accession codes GSE169749 (https://www.ncbi.nlm.nih.gov/geo/query/acc.cgi?acc=GSE169749) and GSE190595 (https://www.ncbi.nlm.nih.gov/geo/query/acc.cgi?acc=GSE190595) for spatial transcriptomics, and GSE163638 (https://www.ncbi.nlm.nih.gov/geo/query/acc.cgi?acc=GSE163638) for scRNAseq. The published datasets used in this study are available under the following accession codes: GSE131032 (https://www.ncbi.nlm.nih.gov/geo/query/acc.cgi?acc=GSE131032) (mouse longitudinal DSS kinetics)10; GSE158702 (https://www.ncbi.nlm.nih.gov/geo/query/acc.cgi?acc=GSE158702) (human scRNAseq)32. The pictures from the Human Protein Atlas are available at: https://www.proteinatlas.org/.

Field-specific reporting

Please select the one below that is the best fit for your research. If you are not sure, read the appropriate sections before making your selection.

[x] Life sciences [ ] Behavioural & social sciences [ ] Ecological, evolutionary & environmental sciences

For a reference copy of the document with all sections, see nature.com/documents/nr-reporting-summary-flat.pdf

Life sciences study design

All studies must disclose on these points even when the disclosure is negative.

Sample size	No sample size calculation was performed. For qPCR validation experiments, we used n=3 mice based on prior publication using comparable methods and based on the distribution of the results (i.e. low SD). For Swiss roll processing for Visium, we used n=1 for d0 and n=2 for d14. We selected the tissue with representation of all anatomical structures usually identified in the colon (for d0 and d14; e.g. ILF) and with some degree of damage (for d14).
Data exclusions	We did not excluded data
Replication	qPCR experiments were performed one time in technical replicate and on three biological replicates (all attempts at replication were successful). Representative pictures from the Human Protein Atlas displayed in Figures 1d, 2c and 2e are representative of 2-12 pictures (all attempts at replication were successful). The spatial transcriptomics data presented in all the Main and Supplementary Figures are generated from n=1 mouse on d0 and n=1 mouse on d14 after DSS treatment. A biological replicate of d14 spatial transcriptomic analysis is presented in Supplementary Figure 8 (the attempt at replication was successful).
Randomization	Animal experiments were age-matched and housed in the same cage prior to DSS administration. Mice were randomly assigned to the treated (i.e. d14) or untreated (i.e. d0) group.
Blinding	Histological assessment of the colonic tissue at d14 was performed blindly by a pathologist. The other results were not performed blindly as they were performed by individual researchers, and they do not encompass subjective measurements.

Reporting for specific materials, systems and methods

We require information from authors about some types of materials, experimental systems and methods used in many studies. Here, indicate whether each material, system or method listed is relevant to your study. If you are not sure if a list item applies to your research, read the appropriate section before selecting a response.

Materials & experimental systems

n/a	Involved in the study	
[x]	[ ]	Antibodies
[x]	[ ]	Eukaryotic cell lines
[x]	[ ]	Palaeontology and archaeology
[ ]	[x]	Animals and other organisms
[x]	[ ]	Human research participants
[x]	[ ]	Clinical data
[x]	[ ]	Dual use research of concern

Methods

n/a	Involved in the study	
[x]	[ ]	ChIP-seq
[x]	[ ]	Flow cytometry
[x]	[ ]	MRI-based neuroimaging

Animals and other organisms

Policy information about studies involving animals; ARRIVE guidelines recommended for reporting animal research

Laboratory animals	Female WT C57BL/6J mice between 6-8 weeks old were purchased from TACONIC. All mice were housed in colony cages in a pathogen-free environment with the temperature maintained at 21–23 °C and relative humidity at 50–60%, and were under a 12 hr light/12 hr dark cycle. All mice were fed ad libitum with standard chow diet.
Wild animals	No wild animals were collected in this study
Field-collected samples	No field collected animals were used in this study
Ethics oversight	All experimental procedures were performed according to national (Sweden) and institutional (Karolinska Institutet) regulations and guidelines. Animals were maintained under specific pathogen-free conditions at AKM animal Facility (Stockholm, Sweden) and handled according to protocols approved by the Stockholm Regional Ethics Committee (ethical permit number: AKM 3227-2017).

Note that full information on the approval of the study protocol must also be provided in the manuscript.
